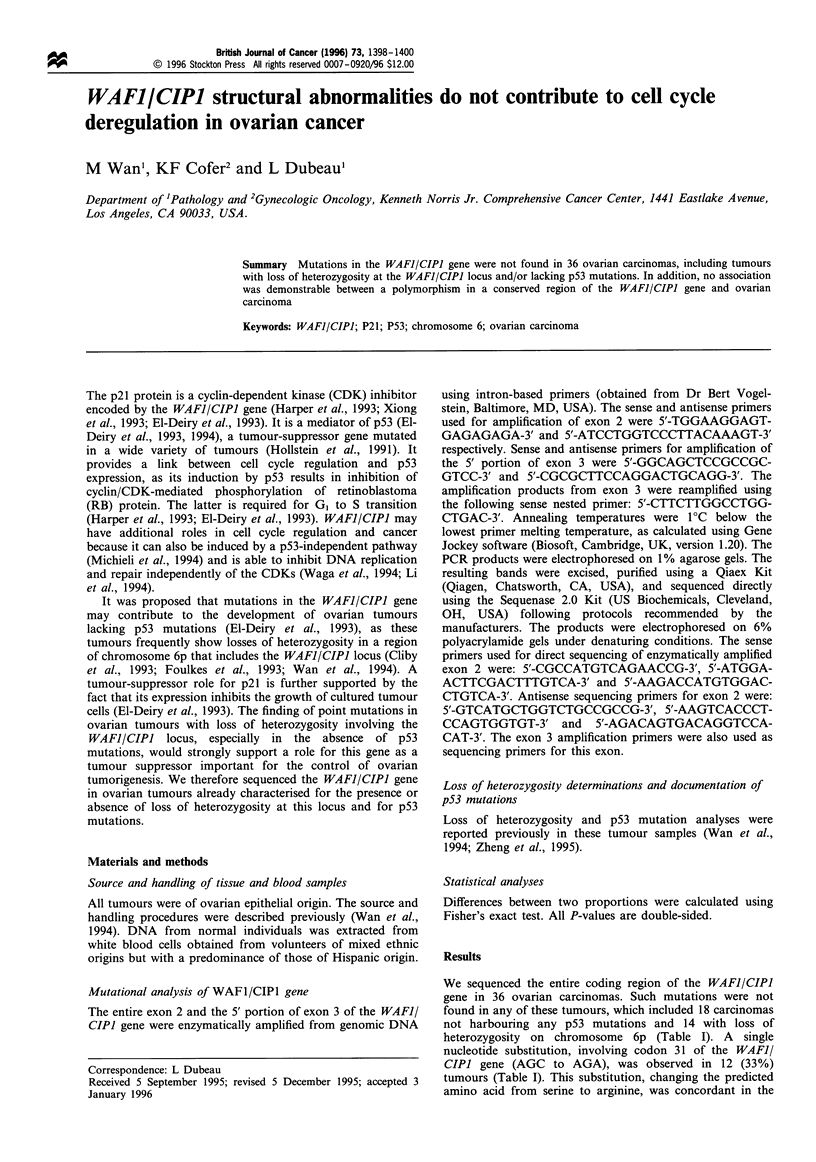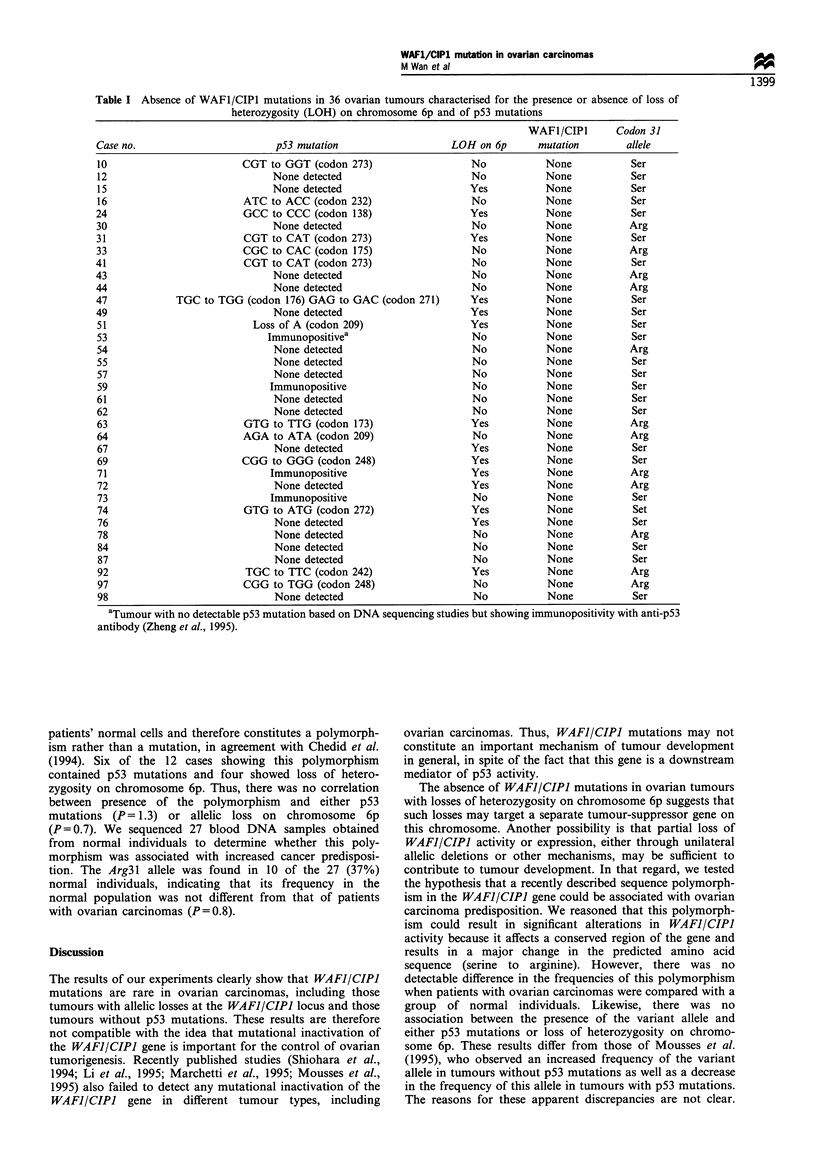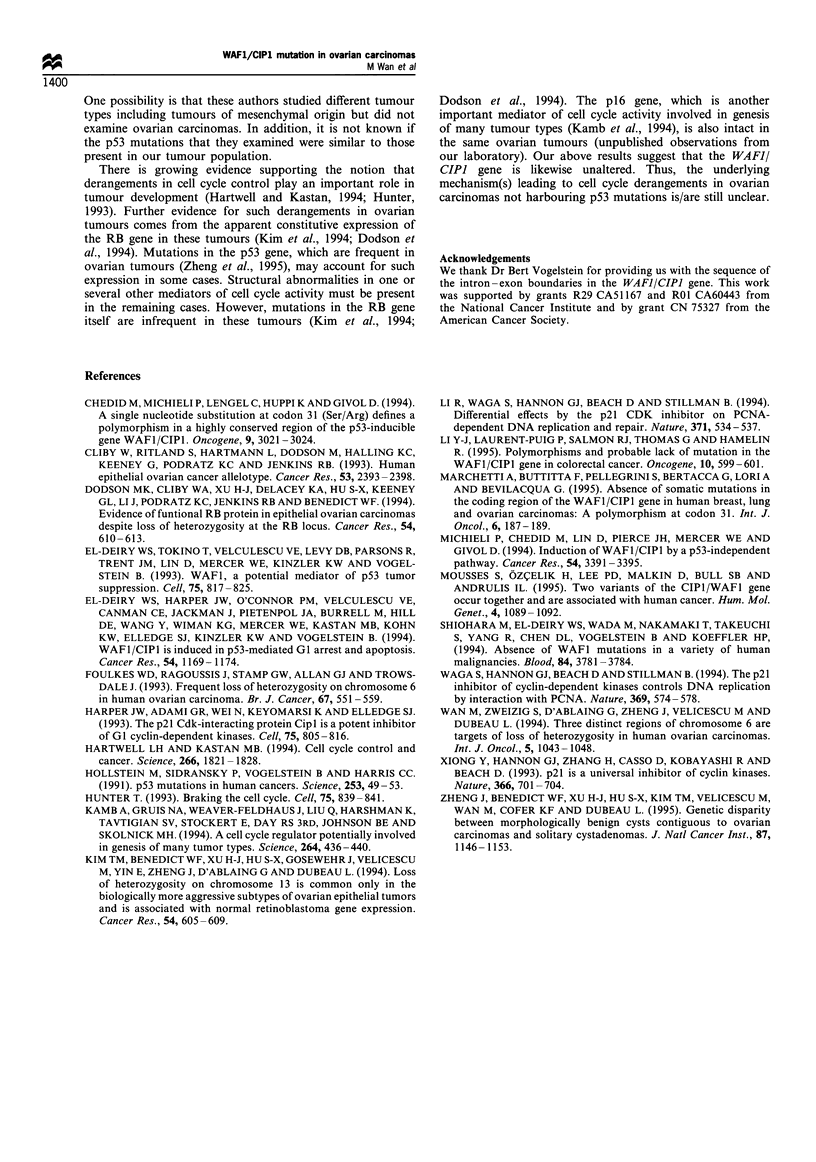# WAF1/CIP1 structural abnormalities do not contribute to cell cycle deregulation in ovarian cancer.

**DOI:** 10.1038/bjc.1996.265

**Published:** 1996-06

**Authors:** M. Wan, K. F. Cofer, L. Dubeau

**Affiliations:** Department of Pathology, Kenneth Norris Jr. Comprehensive Cancer Center, Los Angeles, CA 90033, USA.

## Abstract

Mutations in the WAF1/CIP1 gene were not found in 36 ovarian carcinomas, including tumours with loss of heterozygosity at the WAF1/CIP1 locus and/or lacking p53 mutations. In addition, no association was demonstrable between a polymorphism in a conserved region of the WAF1/CIP1 gene and ovarian carcinoma.


British Journal of Cancer (1996) 73, 1398-1400
© 1996 Stockton Press All rights reserved 0007-0920/96 $12.00

### *WAF1/CIP1* structural abnormalities do not contribute to cell cycle deregulation in ovarian cancer

M Wan[1], KF Cofer[2] and L Dubeau[1]

*Department of [1]Pathology and [2]Gynecologic Oncology, Kenneth Norris Jr. Comprehensive Cancer Center, 1441 Eastlake Avenue, Los Angeles, CA 90033, USA.*

**Summary** Mutations in the *WAF1/CIP1* gene were not found in 36 ovarian carcinomas, including tumours with loss of heterozygosity at the *WAF1/CIP1* locus and/or lacking p53 mutations. In addition, no association was demonstrable between a polymorphism in a conserved region of the *WAF1/CIP1* gene and ovarian carcinoma

**Keywords:** *WAF1/CIP1*; P21; P53; chromosome 6; ovarian carcinoma

The p21 protein is a cyclin-dependent kinase (CDK) inhibitor encoded by the *WAF1/CIP1* gene (Harper *et al.*, 1993; Xiong *et al.*, 1993; El-Deiry *et al.*, 1993). It is a mediator of p53 (El-Deiry *et al.*, 1993, 1994), a tumour-suppressor gene mutated in a wide variety of tumours (Hollstein *et al.*, 1991). It provides a link between cell cycle regulation and p53 expression, as its induction by p53 results in inhibition of cyclin/CDK-mediated phosphorylation of retinoblastoma (RB) protein. The latter is required for $G_1$ to S transition (Harper *et al.*, 1993; El-Deiry *et al.*, 1993). *WAF1/CIP1* may have additional roles in cell cycle regulation and cancer because it can also be induced by a p53-independent pathway (Michieli *et al.*, 1994) and is able to inhibit DNA replication and repair independently of the CDKs (Waga *et al.*, 1994; Li *et al.*, 1994).

It was proposed that mutations in the *WAF1/CIP1* gene may contribute to the development of ovarian tumours lacking p53 mutations (El-Deiry *et al.*, 1993), as these tumours frequently show losses of heterozygosity in a region of chromosome 6p that includes the *WAF1/CIP1* locus (Cliby *et al.*, 1993; Foulkes *et al.*, 1993; Wan *et al.*, 1994). A tumour-suppressor role for p21 is further supported by the fact that its expression inhibits the growth of cultured tumour cells (El-Deiry *et al.*, 1993). The finding of point mutations in ovarian tumours with loss of heterozygosity involving the *WAF1/CIP1* locus, especially in the absence of p53 mutations, would strongly support a role for this gene as a tumour suppressor important for the control of ovarian tumorigenesis. We therefore sequenced the *WAF1/CIP1* gene in ovarian tumours already characterised for the presence or absence of loss of heterozygosity at this locus and for p53 mutations.

### Materials and methods

### *Source and handling of tissue and blood samples*

All tumours were of ovarian epithelial origin. The source and handling procedures were described previously (Wan *et al.*, 1994). DNA from normal individuals was extracted from white blood cells obtained from volunteers of mixed ethnic origins but with a predominance of those of Hispanic origin.

### *Mutational analysis of* WAF1/CIP1 *gene*

The entire exon 2 and the 5′ portion of exon 3 of the *WAF1/CIP1* gene were enzymatically amplified from genomic DNA using intron-based primers (obtained from Dr Bert Vogelstein, Baltimore, MD, USA). The sense and antisense primers used for amplification of exon 2 were 5′-TGGAAGGAGTGAGAGAGA-3′ and 5′-ATCCTGGTCCCTTACAAAGT-3′ respectively. Sense and antisense primers for amplification of the 5′ portion of exon 3 were 5′-GGCAGCTCCGCCGCGTCC-3′ and 5′-CGCGCTTCCAGGACTGCAGG-3′. The amplification products from exon 3 were reamplified using the following sense nested primer: 5′-CTTCTTGGCCTGGCTGAC-3′. Annealing temperatures were 1°C below the lowest primer melting temperature, as calculated using Gene Jockey software (Biosoft, Cambridge, UK, version 1.20). The PCR products were electrophoresed on 1% agarose gels. The resulting bands were excised, purified using a Qiaex Kit (Qiagen, Chatsworth, CA, USA), and sequenced directly using the Sequenase 2.0 Kit (US Biochemicals, Cleveland, OH, USA) following protocols recommended by the manufacturers. The products were electrophoresed on 6% polyacrylamide gels under denaturing conditions. The sense primers used for direct sequencing of enzymatically amplified exon 2 were: 5′-CGCCATGTCAGAACCG-3′, 5′-ATGGAACTTCGACTTTGTCA-3′ and 5′-AAGACCATGTGGACCTGTCA-3′. Antisense sequencing primers for exon 2 were: 5′-GTCATGCTGGTCTGCCGCCG-3′, 5′-AAGTCACCCTCCAGTGGTGT-3′ and 5′-AGACAGTGACAGGTCCACAT-3′. The exon 3 amplification primers were also used as sequencing primers for this exon.

### *Loss of heterozygosity determinations and documentation of p53 mutations*

Loss of heterozygosity and p53 mutation analyses were reported previously in these tumour samples (Wan *et al.*, 1994; Zheng *et al.*, 1995).

### *Statistical analyses*

Differences between two proportions were calculated using Fisher's exact test. All *P*-values are double-sided.

### Results

We sequenced the entire coding region of the *WAF1/CIP1* gene in 36 ovarian carcinomas. Such mutations were not found in any of these tumours, which included 18 carcinomas not harbouring any p53 mutations and 14 with loss of heterozygosity on chromosome 6p (Table I). A single nucleotide substitution, involving codon 31 of the *WAF1/CIP1* gene (AGC to AGA), was observed in 12 (33%) tumours (Table I). This substitution, changing the predicted amino acid from serine to arginine, was concordant in the

Correspondence: L Dubeau
Received 5 September 1995; revised 5 December 1995; accepted 3 January 1996

Table I Absence of WAF1/CIP1 mutations in 36 ovarian tumours characterised for the presence or absence of loss of heterozygosity (LOH) on chromosome 6p and of p53 mutations

| Case no. | p53 mutation | LOH on 6p | WAF1/CIP1 mutation | Codon 31 allele |
|---|---|---|---|---|
| 10 | CGT to GGT (codon 273) | No | None | Ser |
| 12 | None detected | No | None | Ser |
| 15 | None detected | Yes | None | Ser |
| 16 | ATC to ACC (codon 232) | No | None | Ser |
| 24 | GCC to CCC (codon 138) | Yes | None | Ser |
| 30 | None detected | No | None | Arg |
| 31 | CGT to CAT (codon 273) | Yes | None | Ser |
| 33 | CGC to CAC (codon 175) | No | None | Arg |
| 41 | CGT to CAT (codon 273) | No | None | Ser |
| 43 | None detected | No | None | Arg |
| 44 | None detected | No | None | Arg |
| 47 | TGC to TGG (codon 176) GAG to GAC (codon 271) | Yes | None | Ser |
| 49 | None detected | Yes | None | Ser |
| 51 | Loss of A (codon 209) | Yes | None | Ser |
| 53 | Immunopositive[a] | No | None | Ser |
| 54 | None detected | No | None | Arg |
| 55 | None detected | No | None | Ser |
| 57 | None detected | No | None | Ser |
| 59 | Immunopositive | No | None | Ser |
| 61 | None detected | No | None | Ser |
| 62 | None detected | No | None | Ser |
| 63 | GTG to TTG (codon 173) | Yes | None | Arg |
| 64 | AGA to ATA (codon 209) | No | None | Arg |
| 67 | None detected | Yes | None | Ser |
| 69 | CGG to GGG (codon 248) | Yes | None | Ser |
| 71 | Immunopositive | Yes | None | Arg |
| 72 | None detected | Yes | None | Arg |
| 73 | Immunopositive | No | None | Ser |
| 74 | GTG to ATG (codon 272) | Yes | None | Set |
| 76 | None detected | Yes | None | Ser |
| 78 | None detected | No | None | Arg |
| 84 | None detected | No | None | Ser |
| 87 | None detected | No | None | Ser |
| 92 | TGC to TTC (codon 242) | Yes | None | Arg |
| 97 | CGG to TGG (codon 248) | No | None | Arg |
| 98 | None detected | No | None | Ser |

[a]Tumour with no detectable p53 mutation based on DNA sequencing studies but showing immunopositivity with anti-p53 antibody (Zheng *et al*., 1995).

patients' normal cells and therefore constitutes a polymorphism rather than a mutation, in agreement with Chedid *et al*. (1994). Six of the 12 cases showing this polymorphism contained p53 mutations and four showed loss of heterozygosity on chromosome 6p. Thus, there was no correlation between presence of the polymorphism and either p53 mutations ($P=1.3$) or allelic loss on chromosome 6p ($P=0.7$). We sequenced 27 blood DNA samples obtained from normal individuals to determine whether this polymorphism was associated with increased cancer predisposition. The *Arg*31 allele was found in 10 of the 27 (37%) normal individuals, indicating that its frequency in the normal population was not different from that of patients with ovarian carcinomas ($P=0.8$).

### Discussion

The results of our experiments clearly show that *WAF1/CIP1* mutations are rare in ovarian carcinomas, including those tumours with allelic losses at the *WAF1/CIP1* locus and those tumours without p53 mutations. These results are therefore not compatible with the idea that mutational inactivation of the *WAF1/CIP1* gene is important for the control of ovarian tumorigenesis. Recently published studies (Shiohara *et al*., 1994; Li *et al*., 1995; Marchetti *et al*., 1995; Mousses *et al*., 1995) also failed to detect any mutational inactivation of the *WAF1/CIP1* gene in different tumour types, including ovarian carcinomas. Thus, *WAF1/CIP1* mutations may not constitute an important mechanism of tumour development in general, in spite of the fact that this gene is a downstream mediator of p53 activity.

The absence of *WAF1/CIP1* mutations in ovarian tumours with losses of heterozygosity on chromosome 6p suggests that such losses may target a separate tumour-suppressor gene on this chromosome. Another possibility is that partial loss of *WAF1/CIP1* activity or expression, either through unilateral allelic deletions or other mechanisms, may be sufficient to contribute to tumour development. In that regard, we tested the hypothesis that a recently described sequence polymorphism in the *WAF1/CIP1* gene could be associated with ovarian carcinoma predisposition. We reasoned that this polymorphism could result in significant alterations in *WAF1/CIP1* activity because it affects a conserved region of the gene and results in a major change in the predicted amino acid sequence (serine to arginine). However, there was no detectable difference in the frequencies of this polymorphism when patients with ovarian carcinomas were compared with a group of normal individuals. Likewise, there was no association between the presence of the variant allele and either p53 mutations or loss of heterozygosity on chromosome 6p. These results differ from those of Mousses *et al*. (1995), who observed an increased frequency of the variant allele in tumours without p53 mutations as well as a decrease in the frequency of this allele in tumours with p53 mutations. The reasons for these apparent discrepancies are not clear.

One possibility is that these authors studied different tumour types including tumours of mesenchymal origin but did not examine ovarian carcinomas. In addition, it is not known if the p53 mutations that they examined were similar to those present in our tumour population.

There is growing evidence supporting the notion that derangements in cell cycle control play an important role in tumour development (Hartwell and Kastan, 1994; Hunter, 1993). Further evidence for such derangements in ovarian tumours comes from the apparent constitutive expression of the RB gene in these tumours (Kim et al., 1994; Dodson et al., 1994). Mutations in the p53 gene, which are frequent in ovarian tumours (Zheng et al., 1995), may account for such expression in some cases. Structural abnormalities in one or several other mediators of cell cycle activity must be present in the remaining cases. However, mutations in the RB gene itself are infrequent in these tumours (Kim et al., 1994; Dodson et al., 1994). The p16 gene, which is another important mediator of cell cycle activity involved in genesis of many tumour types (Kamb et al., 1994), is also intact in the same ovarian tumours (unpublished observations from our laboratory). Our above results suggest that the *WAF1/CIP1* gene is likewise unaltered. Thus, the underlying mechanism(s) leading to cell cycle derangements in ovarian carcinomas not harbouring p53 mutations is/are still unclear.

**Acknowledgements**

We thank Dr Bert Vogelstein for providing us with the sequence of the intron–exon boundaries in the *WAF1/CIP1* gene. This work was supported by grants R29 CA51167 and R01 CA60443 from the National Cancer Institute and by grant CN 75327 from the American Cancer Society.

### References

CHEDID M, MICHIELI P, LENGEL C, HUPPI K AND GIVOL D. (1994). A single nucleotide substitution at codon 31 (Ser/Arg) defines a polymorphism in a highly conserved region of the p53-inducible gene WAF1/CIP1. *Oncogene*, **9**, 3021–3024.

CLIBY W, RITLAND S, HARTMANN L, DODSON M, HALLING KC, KEENEY G, PODRATZ KC AND JENKINS RB. (1993). Human epithelial ovarian cancer allelotype. *Cancer Res.*, **53**, 2393–2398.

DODSON MK, CLIBY WA, XU H-J, DELACEY KA, HU S-X, KEENEY GL, LI J, PODRATZ KC, JENKINS RB AND BENEDICT WF. (1994). Evidence of funtional RB protein in epithelial ovarian carcinomas despite loss of heterozygosity at the RB locus. *Cancer Res.*, **54**, 610–613.

EL-DEIRY WS, TOKINO T, VELCULESCU VE, LEVY DB, PARSONS R, TRENT JM, LIN D, MERCER WE, KINZLER KW AND VOGELSTEIN B. (1993). WAF1, a potential mediator of p53 tumor suppression. *Cell*, **75**, 817–825.

EL-DEIRY WS, HARPER JW, O'CONNOR PM, VELCULESCU VE, CANMAN CE, JACKMAN J, PIETENPOL JA, BURRELL M, HILL DE, WANG Y, WIMAN KG, MERCER WE, KASTAN MB, KOHN KW, ELLEDGE SJ, KINZLER KW AND VOGELSTEIN B. (1994). WAF1/CIP1 is induced in p53-mediated G1 arrest and apoptosis. *Cancer Res.*, **54**, 1169–1174.

FOULKES WD, RAGOUSSIS J, STAMP GW, ALLAN GJ AND TROWSDALE J. (1993). Frequent loss of heterozygosity on chromosome 6 in human ovarian carcinoma. *Br. J. Cancer*, **67**, 551–559.

HARPER JW, ADAMI GR, WEI N, KEYOMARSI K AND ELLEDGE SJ. (1993). The p21 Cdk-interacting protein Cip1 is a potent inhibitor of G1 cyclin-dependent kinases. *Cell*, **75**, 805–816.

HARTWELL LH AND KASTAN MB. (1994). Cell cycle control and cancer. *Science*, **266**, 1821–1828.

HOLLSTEIN M, SIDRANSKY P, VOGELSTEIN B AND HARRIS CC. (1991). p53 mutations in human cancers. *Science*, **253**, 49–53.

HUNTER T. (1993). Braking the cell cycle. *Cell*, **75**, 839–841.

KAMB A, GRUIS NA, WEAVER-FELDHAUS J, LIU Q, HARSHMAN K, TAVTIGIAN SV, STOCKERT E, DAY RS 3RD, JOHNSON BE AND SKOLNICK MH. (1994). A cell cycle regulator potentially involved in genesis of many tumor types. *Science*, **264**, 436–440.

KIM TM, BENEDICT WF, XU H-J, HU S-X, GOSEWEHR J, VELICESCU M, YIN E, ZHENG J, D'ABLAING G AND DUBEAU L. (1994). Loss of heterozygosity on chromosome 13 is common only in the biologically more aggressive subtypes of ovarian epithelial tumors and is associated with normal retinoblastoma gene expression. *Cancer Res.*, **54**, 605–609.

LI R, WAGA S, HANNON GJ, BEACH D AND STILLMAN B. (1994). Differential effects by the p21 CDK inhibitor on PCNA-dependent DNA replication and repair. *Nature*, **371**, 534–537.

LI Y-J, LAURENT-PUIG P, SALMON RJ, THOMAS G AND HAMELIN R. (1995). Polymorphisms and probable lack of mutation in the WAF1/CIP1 gene in colorectal cancer. *Oncogene*, **10**, 599–601.

MARCHETTI A, BUTTITTA F, PELLEGRINI S, BERTACCA G, LORI A AND BEVILACQUA G. (1995). Absence of somatic mutations in the coding region of the WAF1/CIP1 gene in human breast, lung and ovarian carcinomas: A polymorphism at codon 31. *Int. J. Oncol.*, **6**, 187–189.

MICHIELI P, CHEDID M, LIN D, PIERCE JH, MERCER WE AND GIVOL D. (1994). Induction of WAF1/CIP1 by a p53-independent pathway. *Cancer Res.*, **54**, 3391–3395.

MOUSSES S, ÖZÇELIK H, LEE PD, MALKIN D, BULL SB AND ANDRULIS IL. (1995). Two variants of the CIP1/WAF1 gene occur together and are associated with human cancer. *Hum. Mol. Genet.*, **4**, 1089–1092.

SHIOHARA M, EL-DEIRY WS, WADA M, NAKAMAKI T, TAKEUCHI S, YANG R, CHEN DL, VOGELSTEIN B AND KOEFFLER HP, (1994). Absence of WAF1 mutations in a variety of human malignancies. *Blood*, **84**, 3781–3784.

WAGA S, HANNON GJ, BEACH D AND STILLMAN B. (1994). The p21 inhibitor of cyclin-dependent kinases controls DNA replication by interaction with PCNA. *Nature*, **369**, 574–578.

WAN M, ZWEIZIG S, D'ABLAING G, ZHENG J, VELICESCU M AND DUBEAU L. (1994). Three distinct regions of chromosome 6 are targets of loss of heterozygosity in human ovarian carcinomas. *Int. J. Oncol.*, **5**, 1043–1048.

XIONG Y, HANNON GJ, ZHANG H, CASSO D, KOBAYASHI R AND BEACH D. (1993). p21 is a universal inhibitor of cyclin kinases. *Nature*, **366**, 701–704.

ZHENG J, BENEDICT WF, XU H-J, HU S-X, KIM TM, VELICESCU M, WAN M, COFER KF AND DUBEAU L. (1995). Genetic disparity between morphologically benign cysts contiguous to ovarian carcinomas and solitary cystadenomas. *J. Natl Cancer Inst.*, **87**, 1146–1153.